# Platelets as Modifiers of Clinical Phenotype in Hemophilia

**DOI:** 10.1100/tsw.2006.133

**Published:** 2006-06-14

**Authors:** Donald L. Yee

**Affiliations:** Department of Pediatrics, Hematology-Oncology Section and Department of Medicine, Thrombosis Research Section, Baylor College of Medicine, Houston, TX, USA

**Keywords:** hemophilia, platelets, thrombin generation, phenotype, procoagulant

## Abstract

Platelets occupy a central role in the maintenance of hemostasis by adhering to sites of vascular injury and facilitating thrombin generation, which leads to the formation of a fibrin clot. Patients with hemophilia exhibit defective thrombin generation secondary to reduced plasma factor concentrations, which can lead to excessive and sometimes life-threatening bleeding. Individuals differ greatly with respect to platelet function and platelets from different individuals differ inherently in their ability to enact thrombin generation, the key coagulative process that is deficient in hemophilia. Similarly, some patients with hemophilia seem to bleed less often than others despite exhibiting similar plasma factor levels. The biologic factors that underlie this phenotypic variability remain poorly understood, but evidence is reviewed supporting a role for platelets and platelet-related factors in modifying bleeding tendency in patients with hemophilia and potential directions for further clinical research in this area are discussed.

## HEMOPHILIA

Hemophilia A and hemophilia B are X-linked, congenital, bleeding disorders that result from decreased or absent production of either factor VIII (A) or factor IX (B). They are the two most common inherited forms of coagulation protein deficiency, estimated to present in 1 in 5,000 (A) to 20,000 (B) live male births[1]. The factor deficiency results in reduced generation of activated factor X at the platelet surface, which in turn leads to reduced thrombin generation (Fig. 1, panel C). As such, hemophilia is generally regarded as a *coagulation disorder*, and the contribution of cellular blood components, such as platelets, to these diseases has not been widely considered.

Affected individuals demonstrate variable degrees of bleeding tendency (clinical phenotype) that generally correlate with the degree of factor deficiency[1]. In practice, patients are categorized as having mild, moderate, or severe disease based on laboratory assay determinations of baseline circulating factor levels. Patients with “severe” hemophilia, defined as plasma factor levels <1% of normal[2], generally exhibit the most severe bleeding tendency, manifest as spontaneous bleeding into joints or muscles, or bleeding after minimal trauma; they often require treatment with exogenous factor in order to control or prevent bleeding. However, some patients with “severe” factor deficiency appear to have atypically “mild”

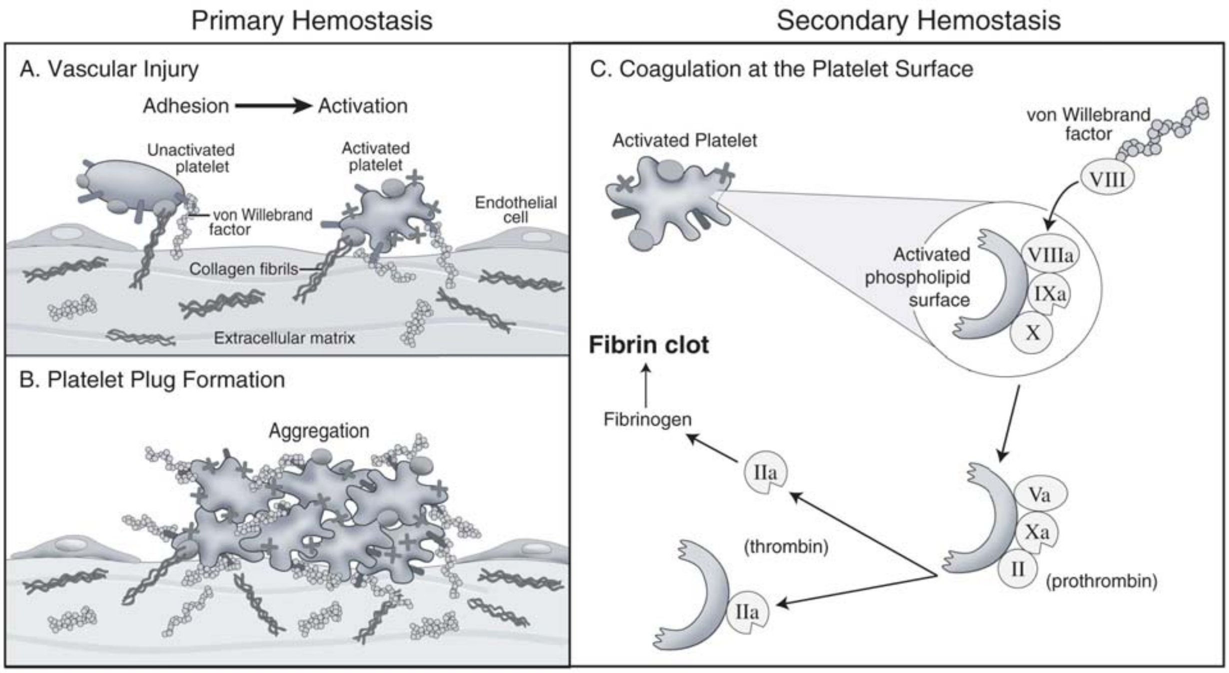

**FIGURE 1.** Overview of hemostasis**.** With vessel injury (panel A), circulating unactivated platelets tether to exposed subendothelial matrix via specific platelet receptors (circles and rods). With progressive adhesion, platelets undergo activation, shape change, and exposure of other activated receptors (crosses), culminating in formation of the platelet plug (panel B). At the activated platelet surface (crescentic forms, panel C), phosphatidylserine (a coagulation cofactor) is preferentially exposed at the outer membrane leaflet, thereby potentiating the activation of factor X to factor Xa and factor II (prothrombin) to factor IIa (thrombin). Thrombin is the critical enzyme that allows formation of the fibrin clot. Factors VIII and IX, the proteins that are deficient in hemophilia A and hemophilia B, respectively, are cofactor (in its activated form, factor VIIIa) and zymogen (factor IXa is the active enzyme) for the initial reaction depicted in panel C. Factor VIII normally circulates in the plasma bound to von Willebrand factor. Roman numerals signify coagulation proteins, functioning as either zymogens (circles), enzymes (excised circles), or cofactors (ovals).

disease, with significantly reduced frequencies of spontaneous bleeding and lower requirements for factor infusions. The terms “mild severe” and “severe severe” have been used to classify patients who demonstrate disparate bleeding tendencies despite equally severe factor deficiency as measured by laboratory assay[3]. Such heterogeneity in phenotype is frequently observed in clinical practice[4,5,6,7] (Fig. 2).

The existence of varied clinical phenotypes, even among individuals with the same molecular defect[8,9], implicates other sources of disease modulation besides mutations in the factor VIII or IX gene, including genetic modifiers. Several case-control studies have examined the impact of various heritable prothrombotic risk factors (most commonly factor V Leiden) on bleeding tendency in order to determine why certain patients seem to bleed less often than others[3,8,9,10,11,12,13,14]. However, the conclusions have generated controversy as some have found associations, others have not, and all studies have been small and varied in design[15,16]. Even in the studies that found associations, most “mild severe” hemophilia patients had no identifiable factor that would be expected to attenuate bleeding, so that most phenotypic variability must be due to other, as yet unidentified, factors. Inherent hemostatic properties that vary significantly between individuals are attractive candidates for further study of the basis for this phenotypic variability.

## THE ROLE OF PLATELETS IN HEMOSTASIS AND THROMBIN GENERATION

Circulating platelets fulfill many critical functions in the maintenance of hemostasis, including adhesion to sites of vascular injury, activation that amplifies the response to injury, secretion of chemical mediators

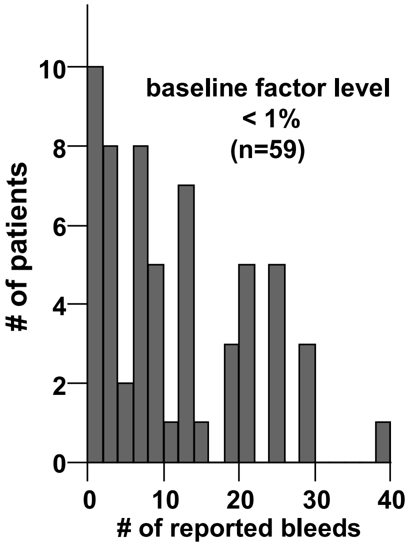

**FIGURE 2.** Variation in bleeding frequency among severe hemophilia A patients during a 6-month period**.** Histogram displaying broad range of bleeding episodes reported (on Universal Data Collection) by a cohort of 59 patients followed at Gulf States Hemophilia Treatment Center in Houston, TX. Each of these patients received only episodic (no prophylaxis) factor replacement. Data courtesy of Dr. W. Keith Hoots.

of the hemostatic response, and aggregation via fibrinogen binding[17]. After vascular injury, these processes initially lead to the formation of a platelet plug, and constitute *primary hemostasis*[18]. Platelets also play a central role in *secondary hemostasis*[19,20] (i.e., *coagulation*), the series of catalytic reactions involving plasma serine proteases and their cofactors that culminates in thrombin generation and the formation of a stable fibrin clot at the site of the platelet plug (Fig. 1). Platelets potentiate secondary hemostasis by providing a phospholipid (cofactor) surface on which several key coagulation reactions can take place[21]. For example, the activated platelet surface accelerates the factor VIIIa/IXa-mediated conversion of factor X to Xa by $2 \times 10^7$-fold[22] and the factor Va/Xa-mediated conversion of prothrombin to thrombin by 300,000-fold[23,24].

Platelet function (primary hemostasis) and coagulation reactions leading to thrombin generation (secondary hemostasis) are, thus, tightly interwoven processes. During platelet activation, multiple events occur that constitute the platelet procoagulant response, including (1) translocation of anionic phospolipids (primarily phosphatidylserine) from the inner to the outer leaflet of the platelet membrane, providing cofactors for enzymatic reactions in the coagulation sequence[25]; (2) increased binding of activated coagulation factors to the platelet surface[26,27,28], facilitating enzyme-substrate interactions; and (3) membrane vesiculation and generation of procoagulant microparticles that aid in the initiation and propagation of coagulation reactions[29]. Platelet surface receptors appear to facilitate this platelet procoagulant response[30,31]. As up to a fivefold variation in platelet procoagulant activity has been observed between healthy subjects[32,33], it has been suggested that structural differences in such receptors may underlie these functional differences between individuals[31].

The importance of platelets for adequate thrombin generation and clot formation has been well established in a variety of model systems, including studies of patients with hemophilia. Studies utilizing antiplatelet agents demonstrate that thrombin generation varies directly with platelet function[34,35,36]. Moreover, in hemophilia patients, the modulating effect of platelets on thrombin generation increases at lower factor levels[37], highlighting the potential role that inherent, interindividual differences in platelet function may play in modifying the clinical phenotype in such patients. Marked variation in thrombin-generating capacity has been observed between hemophilia patients with similar baseline factor levels[38,39]; although one study observed a correlation between the clinical and laboratory phenotypes[39], the other did not[38]. The specific contribution of platelets to the differences in thrombin generation between patients requires further study. Thromboelastography experiments have also shown substantial variation in hemostatic profiles between different hemophilia patients with the same factor level[40,41]. Studies of hemophilic subjects using this assay system confirm that clot formation depends greatly on platelet number[42] and function[43]; decrements in either of these results in corresponding deficiencies in the thromboelastographic profile (although correlative studies linking to clinical bleeding risk are limited).

## A POTENTIAL LINK BETWEEN PLATELET AND HEMOPHILIA PHENOTYPES

When platelet function is measured *in vitro*, substantial interindividual variability is observed, even in healthy individuals[44,45,46,47] (Fig. 3). Given the important contributions platelets make to hemostasis in general, and to thrombin generation in particular, such differences could be expected to modulate an individual's predisposition to thrombosis or to bleeding. For example, platelets with lower thresholds for activation may exhibit procoagulant activity leading to thrombin generation at correspondingly lower levels of stimulation, thus resulting in less severe bleeding tendency for a given factor level in patients with hemophilia. Numerous studies have linked increased platelet function to increased *thrombotic* risk[48,49,50,51,52,53,54,55]. However, little consideration has been given to the modifying effects of functional platelet variation on *hemorrhagic* disorders (with the obvious exception of primary platelet disorders). Although a few reports address platelet receptor gene polymorphisms (and consequent differences in platelet function) as important risk factors for bleeding severity in von Willebrand disease[56,57,58] and other clinical settings[59,60], no such data have been reported for coagulation disorders, such as hemophilia.

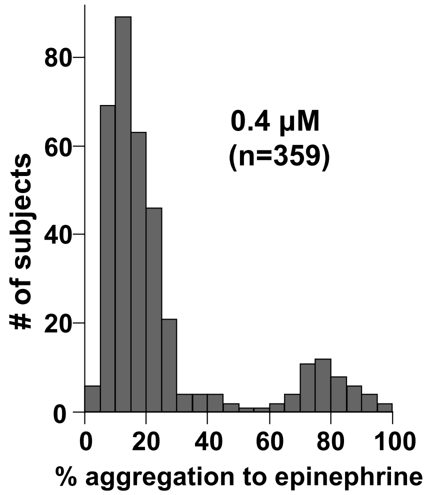

**FIGURE 3.** Range of aggregation response to epinephrine among healthy individuals. Histogram displaying broad range of platelet aggregation response to 0.4 µM/l epinephrine. Use of this low, "submaximal" epinephrine concentration highlights a minority of subjects who demonstrate a relatively hyperreactive response (>60% aggregation) compared to the majority who do not (<40% aggregation). At higher epinephrine concentrations more typically used in clinical applications of aggregometry, neither this hyperreactive minority nor the broad, heterogeneous distribution of platelet responses is appreciated. This research was originally published in *Blood*. Yee et al. (2005) Aggregometry detects platelet hyperreactivity in healthy individuals. *Blood* 106, 2723–2729. © The American Society of Hematology.

However, some previous work suggests an important role for platelets in the modulation of bleeding phenotype in hemophilia. Prolonged bleeding time has been linked to increased bleeding and factor use among patients with hemophilia[61], implicating primary hemostasis in disease modulation. Furthermore, in a case-control study (total n = 32) comparing "mild" vs. "severe" phenotypes among hemophilic patients without detectable factor levels (biochemically severe), the "mild" group exhibited higher levels of platelet coagulant activity than the "severe"[62]. This study by Walsh et al. was one of the first to examine factors underlying phenotypic variability in severe hemophilia and, as such, it utilized several early methods of examining platelet coagulant activity. In the more than 30 years since this important study was reported, no published studies have pursued this line of inquiry further, yet more specific assay methods that examine the most physiologically relevant aspects of platelet procoagulant activity have been developed[32,63]; such methods should be applied to confirm and extend this important work in larger clinical studies.

Procoagulant microparticles are increasingly recognized as important mediators of hemostasis and thrombosis[64,65]. Higher microparticle levels led directly to improved hemostasis in a mouse model of hemophilia A[66]. Furthermore, in the only published study examining the clinical significance of procoagulant microparticles in patients with hemostatic disorders, elevated platelet-derived microparticle levels were associated with decreased bleeding symptoms in patients with idiopathic thrombocytopenic purpura[67]. Most recent studies on procoagulant microparticles have focused on their role in thrombus formation, however, these reports provide indirect evidence of microparticles' potential role in hemostatic protection as well. Platelet-derived microparticles demonstrate at least a fourfold variation in healthy individuals[68] and even greater heterogeneity among hemophilia patients[69]; such interindividual variability further supports study of microparticles in disease modulation of hemophilia.

## CONCLUDING REMARKS

Approximately 400,000 individuals worldwide carry the diagnosis of hemophilia[70], yet the basis for much of the phenotypic variability observed in this disorder remains poorly understood. Further work is required to elucidate relevant risk factors and mechanisms underlying these differences in bleeding tendency; platelets (and platelet-related factors) are a promising direction for this research by virtue of their primary role in hemostasis and the degree of variability in platelet function seen between different individuals. As some hemophilic individuals have a more severe clinical phenotype than others and thus require greater utilization of medical resources (e.g., factor use, hospitalizations), identifying risk factors that predict bleeding could enable more rational resource allocation. Identification of disease-modifying, platelet-related factors in hemophilia may also suggest potential alternative targets for preventive and therapeutic intervention and guide future studies into specific mechanisms of platelet physiology relevant to the interaction between platelets and coagulation proteins.

## ACKNOWLEDGMENTS

The author is supported by career development awards from the NIH/NHLBI (HL81539) and the National Hemophilia Foundation.
